# Tumors of the Tongue

**Published:** 1875-11

**Authors:** M. Molliere


### ARTICLE V.

### *On Tumours of the Tongue.*

M. Molliere, Surgeon to the Hotel-Dieu at Lyons, makes the following observations upon some rare tumours of the tongue, (*Progres Medicale, No.* 1, 1875.)

*Hydatid Cyst of the Tongue.*—L. S., aged twenty-four, a schoolmaster, entered the Hotel-Dien, at Lyons, on September 18, 1874. This young man, who seemed to be of a robust constitution, dated his malady from the preceding March. A pimple then appeared upon the tongue, followed in a few days by violent pain. The pimple was then about the size of a pea, but rapidly increased. Inflammatory symptoms of great intensity supervened. The tongue acquired an enormous size, and for fifteen days the patient could swallow no food. When this inflammation ceased, he found the tumour of the same size as before. It was indolent, but it continued to enlarge, so that after some time he had much difficulty in performing the duties of his calling. When he entered the hospital a round and very hard tumour, about the size of a nut, was seen on the right border of the tongue, its greatest prominence being towards the upper surface. This tumour was well defined, regularly rounded, and indolent. The tongue was siezed with a napkin, and drawn with a certain degree of force upwards and out of the mouth. It was thus rendered motionless, and in this way fluctuation was very distinctly observed. The tumour was diagnosed to be a cyst. On September 12 it was operated on. The point of the tongue was siezed with sharp forceps, and drawn outwards. A longitudinal incision was made in the dorsum, and the dissection carried on. The tumour adhered closely to the lingual tissues, and was deeply imbedded in the muscles among which it had its origin. There was also a deep adhesion to the ranine artery, which was opened quite at the base of the tongue When the tumour was removed, it was very difficult to tie the artery. The wound was united by fine stitches of metallic suture. The patient was put to bed, and during the day was ordered gargles of iced and medicated water. Next day there was slight swelling. The tongue was as large as it had been before the operation, of a blue colour, and ecchymosed. There was no fever, and the temperature throughout the whole treatment never reached 39° Cent. (100.2° Fahr.) On the fourth day the stitches were removed. The clots which had accumulated were pressed out of the cavity, and on September 20 the patient left the hospital with only a small linear wound and slight swelling? He could speak freely, and had no pain. When the tumour was examined, a transparent vesicle with very thin walls was found within. It contained a small opaque point. This point was submitted to microscopic examination, and the parasite was discovered with its suckers and crown of hooklets, thus demonstrating the tumour to be an hydatid cyst of the tongue.

M. Molliere's reason for publishing this case is not so much the rarity of hydatid cysts of the tongue, for this rarity is only comparative ; but because of the circumstances which accompanied it. The parasite must have entered the tongue during mastication, and the inflammatory symptoms were probably due to it. In the first place it acted as a foreign body, and we must suppose that the inflammation ceased when the parasitical cyst had formed.

He calls attention particularly to the method he employed to demonstrate the fluctuation. Its value cannot be appreciated without a trial. It is, in fact, the only way in which the organ can be rendered motionless. The therapeutics treatment, also, he considers to have been the most certain and the most rational. Are we not certain by complete excision thoroughly to extirpate the evil? Total ablation also has the advantage of being much quicker than the other methods—injections, partial excisions, or cauterizations. Finally, he says, the only danger to be feared—that of hemorrhage—is guarded against by the use of sutures. His observations upon this point are, in his opinion, as conclusive as possible, since the suture succeeded in arresting the flow of blood, even when it arose from a wound in the principal artery of the organ. It appears to him that the suture is not sufficiently used as a hæmostatic remedy in operations upon the tongue. He speaks, however only of the metallic suture. It has the immense advantage over sutures of vegetable material, that it covers the affected region with small sharp points, so that the patient instinctively keeps the tongue at rest.

*Lipoma of the Tongue.*—The patient was an old man, aged sixty. He had on the left side of his tongue a little tumour, of the size of a haricot bean. This tumour, which was seen under the stretched and transparent mucous membrane, was sofa and of a yellow colour. The patient could give no information as to its origin or course. M. Molliere at first believed it to be an abscess or a syphilitic gumma. But there was nothing in the man's previous history to support this idea; and as the author could not dsscover any fluctuation, he concluded that it was a solid-tumour, developed probably in the glands. As it continued to enlarge, he proposed its ablation and proceeded as follows: A hole was made in a piece of cardboard through which the patient passed his tongue. An assistant held it, while the surgeon laid hold of the tumour with Guersant's hemorrhoidal forceps, which had first been made red hot

After eight days the patient left the hospital cured. Histological examination proved that the tumour was a lipoma. Similar small tumors were seen at the base of the organ but as they caused no inconvenience, they were not touched. Lipoma of the tongue is very rare. M. Molliere calls special attention, however, to the method which he used in removing this tumour. It is expeditious, attended by but little pain, and is free from all danger of hemorrhage. He says he has had recourse to it in other instances to remove small cancers situated on the point or edge of the tongue; and thinks that it ought to be substituted for the knife or the ecraseur in all cases to which it is applicable.—*London Medical Record.*

---

